# Use of a Preclinical Natural Transmission Model to Study Antiviral Effects of a Carbohydrate-Binding Module Therapy against SARS-CoV-2 in Hamsters

**DOI:** 10.3390/v15030725

**Published:** 2023-03-11

**Authors:** Daniel Knott, Rachel Fell, Jane A. Potter, Samantha Yuille, Franscisco J. Salguero, Victoria A. Graham, Roger Hewson, David Howat, Stuart D. Dowall

**Affiliations:** 1 UK Health Security Agency (UKHSA), Salisbury SP4 0JG, UK; daniel.knott@ukhsa.gov.uk (D.K.); rachel.fell@ukhsa.gov.uk (R.F.); javier.salguero@ukhsa.gov.uk (F.J.S.); victoria.graham@ukhsa.gov.uk (V.A.G.); roger.hewson@ukhsa.gov.uk (R.H.); 2 Pneumagen Ltd., Kinburn Castle, Doubledykes Road, St Andrews, Fife KY16 9DR, UK; jane.potter@pneumagen.com (J.A.P.); samantha.yuille@pneumagen.com (S.Y.); david.howat@pneumagen.com (D.H.)

**Keywords:** coronavirus, broad spectrum, efficacy, prophylaxis

## Abstract

The emergence of severe acute respiratory syndrome coronavirus (SARS-CoV-2) and its expansion to a worldwide pandemic resulted in efforts to assess and develop interventions to reduce the disease burden. Despite the introduction of vaccine programmes against SARS-CoV-2, global incidence levels in early 2022 remained high, demonstrating a need for the development of physiologically relevant models, which are essential for the identification of alternative antiviral strategies. The hamster model of SARS-CoV-2 infection has been widely adopted due to similarities with humans in terms of host cell entry mechanism (via ACE2), and aspects of symptomology and virus shedding. We have previously described a natural transmission hamster model that better represents the natural course of infection. In the present study, we have conducted further testing of the model using the first-in-class antiviral Neumifil, which has previously shown promise against SARS-CoV-2 after a direct intranasal challenge. Neumifil is an intranasally delivered carbohydrate-binding module (CBM) which reduces the binding of viruses to their cellular receptor. By targeting the host cell, Neumifil has the potential to provide broad protection against multiple pathogens and variants. This study demonstrates that using a combination of a prophylactic and therapeutic delivery of Neumifil significantly reduces the severity of clinical signs in animals infected via a natural route of transmission and indicates a reduction of viral loads in the upper respiratory tract. Further refinements of the model are required in order to ensure the adequate transmission of the virus. However, our results provide additional data to the evidence base of Neumifil efficacy against respiratory virus infection and demonstrate that the transmission model is a potentially valuable tool for testing antiviral compounds against SARS-CoV-2.

## 1. Introduction

The global pandemic of severe acute respiratory syndrome coronavirus 2 (SARS-CoV-2) resulted in international efforts to combat the threat to human health. The development of vaccines has provided an immense contribution, but case levels remain stubbornly high. The limitations of vaccines include efficacy waning over time, underscoring the requirement for booster doses, and breakthrough infections, including the emergence of multiple variants that reduce vaccine effectiveness [1,2]. The need for effective therapies remains a cornerstone in the ability to bring the pandemic under control and contribute to an arsenal of countermeasures against the virus.

To test interventions in a whole-body system, there is currently no alternative to the use of animal models. For SARS-CoV-2 studies, a range of laboratory animals have been used including mice, hamsters, rats, ferrets and non-human primates [3]. Syrian hamsters have been widely used for COVID-19 research due to their susceptibility to SARS-CoV-2, and active transmission via aerosol and lung pathology resembling human infection [4]. Whilst intranasal delivery of the virus has been widely used for SARS-CoV-2 challenge studies [5], to refine the Syrian hamster model we have developed a natural transmission caging system, where donor animals challenged with SARS-CoV-2 are housed in a central cage and two adjacent cages house recipient animals, with airflow drawing across from the donor animal cage to the side cages [6]. Other natural transmission models for SARS-CoV-2 have also been reported. One of the first publications was where wire cages were used to physically separate hamsters at least 1.8 cm apart within the same cage [7]. Other approaches use a system connecting two cages, with infected animals in one and naïve animals in the other [8]. Alternatively, separation using a double dividing layer of 5 cm but allowing airflow within a ventilated cage has been used [9]. These differ to our approach, as only one test group can be studied at a time. The advantage of having two cages of naïve animals alongside a central cage with an infected donor animal is that is allows control over differences in viral shedding kinetics of individual hamsters. Whilst direct transmission models—where a challenged animal is housed alongside test animals—have shown protective effects for novel COVID-19 vaccine candidates [10], the route of exposure may not solely be through airborne particles.

Multiple licensed therapies have been identified as having positive effects against COVID-19, including those identified through the high-profile RECOVERY clinical trial conducted in the UK [11]. However, newly developed compounds are also rapidly being developed and evaluated. Whilst some approaches target the virus, others target cellular factors essential for virus infection and replication, a strategy which limits the ability of the virus to acquire resistance [12]. One such host-targeted approach is to use bacterially derived carbohydrate-binding modules (CBMs) with high-affinity binding to glycans that can mask cellular receptors and prevent viral attachment [13]. *Sp2*CBMTD is a multivalent form of a sialic acid binding CBM, from the family 40 domain of *Streptococcus pneumoniae* neuraminidase A sialidase. *Sp2*CBMTD has demonstrated efficacy against influenza virus activity in murine models [12,13] and has been modified to reduce predicted immunogenicity in humans whilst retaining ligand binding specificity and affinity in a first-in-class CBM therapy termed Neumifil when administered intranasally.

We have previously reported that Neumifil confers protective effects against an intranasal challenge inoculum of SARS-CoV-2 in the Syrian hamster preclinical model [14]. Within this report, we have assessed the natural transmission model in the context of antiviral agent testing. The model in its current set-up could be used to demonstrate the efficacy of Neumifil against SARS-CoV-2, and further improvements that can be made to increase the usefulness of the model were identified.

## 2. Materials and Methods

### 2.1. Ethical Statement

All experimental protocols with animals were undertaken according to the United Kingdom Animals (Scientific Procedures) Act 1986, with studies conducted under the authority of a UK Home Office approved project licence. The experimental protocols were approved by ethical review at Public Health England (PHE) by the Animal Welfare and Ethical Review Body (AWERB) on 15 July 2021 (Approval Code: PPL PDC57C033). This research is reported in accordance with the ARRIVE guidelines (https://arriveguidelines.org, accessed on 1 March 2022). Prior to the start of the study, humane clinical endpoints were set which consisted of 20 % weight loss, compared with baseline; inactivity/immobility; neurological signs; or on the advice of severe disease from the Named Animal Care and Welfare Officer (NACWO).

### 2.2. Animals

Twenty-four Golden Syrian hamsters aged 7–10 weeks on arrival (mean weight 132.4 g, range 113–154 g) were obtained from a UK Home Office accredited facility (Envigo RMS UK Ltd., Oxford, UK). An equal number of male and female animals were used and animals were randomly assigned to groups. Hamsters were housed in cages designed in accordance with the requirements of the UK Home Office Code of Practice for the Housing and Care of Animals Used on Scientific Procedures (1986). Throughout the course of the study, animals were single housed to reduce confounding factors caused by intra-cage virus exposure seen with group housing. During procedures with SARS-CoV-2, housing and husbandry took place within a flexible-film isolator within a Containment Level 3 facility.

**Figure 1:**
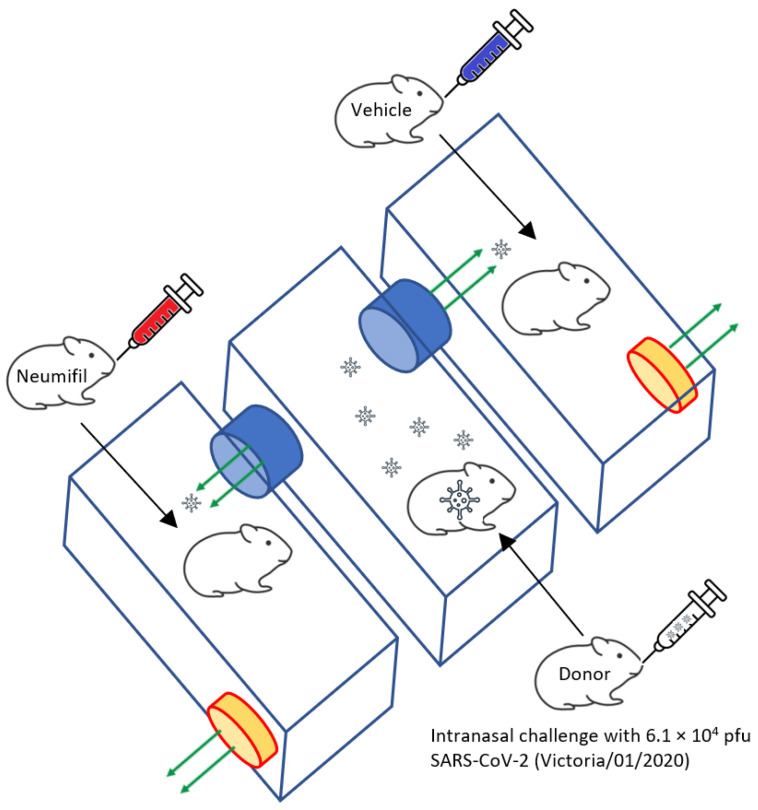
Diagrammatic representation of natural transmission cage layout. Donor animals were challenged with SARS-CoV-2 and placed in the centre cage, with recipient animals receiving Neumifil or vehicle housed in adjacent cages. Fans on the side of the outer two cages allowed airflow to flow across from the centre cage.

To assess the effects of Neumifil, a natural transmission cage system was used, as previously reported [6], with donor animals in the central cage and recipient animals receiving either Neumifil or vehicle alone in the two adjacent cages (Figure 1). Donor hamsters were challenged with 6.1 × 10^4^ plaque-forming units of SARS-CoV-2 (strain Victoria/01/2020 [15]) via intranasal inoculation (100 µL per nare; 200 µL total). Recipient animals were intranasally administered Neumifil (9.4 mg/mL) or vehicle (comprised of Neumifil formulation buffer) in a volume of 50 µL per nare the day before being housed adjacent to the donor animal, and then this was repeated on days 1, 3 and 5 thereafter. During intranasal deliveries, animals were under isoflurane sedation. Animals were weighed daily and clinical scores assessed twice a day by an experienced handler who was blinded as to which recipient animal received Neumifil and which received vehicle. Each clinical sign was assigned a numerical value (2, ruffled fur; 3, wasp-waisted, lethargy, arched; 5, laboured breathing) which were added to derive a score at each monitoring timepoint. The donor animals were euthanised six days post-challenge and the recipient animals were euthanised after a further eight days via anaesthetisation with isoflurane followed by a lethal dose of sodium pentobarbitone.

### 2.3. Sampling and Analysis

One day after the challenge of the donor animals and every other day thereafter, throat swabs were taken via a flocked mini-tip swab and placed into Virocult universal transport medium (MW951T, Medical Wire & Equipment Co Ltd., Corsham, UK). At necropsy, a sample of lung was placed into a PreCellys tube containing ceramic beads and homogenised using a PreCellys21 homogeniser (Stretton Scientific, Alfreton, UK). Following inactivation with either AVL (throat swab) or RLT (lung homogenate) buffer (Qiagen, Manchester, UK), RNA was extracted using a BioSprint One-For-All Vet Kit (Indical, Leipzig, Germany) on a Kingfisher Flex Platform (Thermo-Fisher, Loughborough, UK). Reverse transcription-quantitative polymerase chain reaction (RT-qPCR) of the nucleocapsid gene was used to determine viral loads, as previously described [16].

At necropsy, the left lung and nasal turbinates were collected into 10% neutral-buffered formalin prior to processing, as previously described [16]. Gross changes were observed after haematoxylin and eosin (H&E) staining. In addition, the presence of viral RNA was detected in histological specimens using the RNAscope technique with a V-nCoV2019-S probe (848561, Advanced Cell Diagnostics, Newark, CA, USA). All histological evaluations were undertaken by a qualified veterinary pathologist blinded to the study.

### 2.4. Statistical Analysis

Statistical analyses were performed to assess group differences in weight, clinical score, viral load and histological findings using MiniTab, v.16.2.2 (Minitab Inc, State College, PA, USA). A non-parametric Mann–Whitney statistical test was applied to ascertain significance between groups, with a significance level below 0.05 being considered significant.

## 3. Results

**Figure 2:**
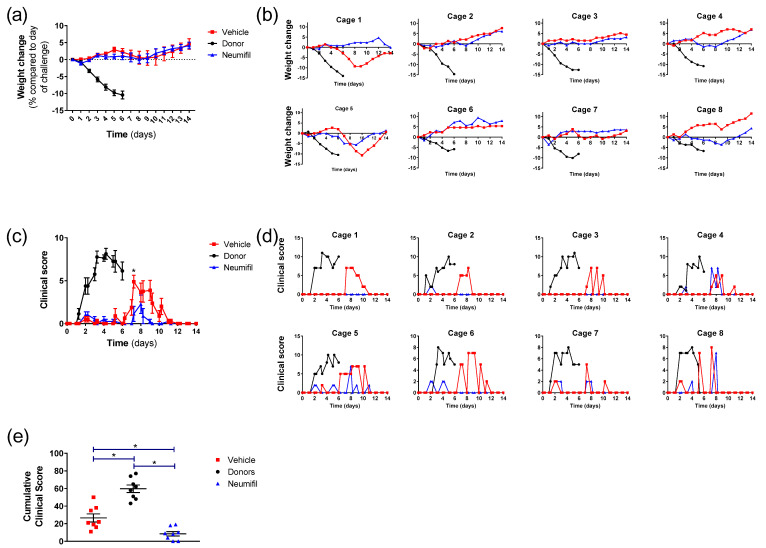
Weight changes and clinical scores in donor animals and recipient animals adjacently housed receiving either Neumifil or vehicle. (**a**,**b**) Weight change represented as percentage change compared to the day of challenge/study start. (**c**,**d**) Clinical scores recorded, with each score assigned a numerical value and added to provide a total score for each specific timepoint. (**a**,**c**) Grouped data from *n* = 8 animals/group. Lines show mean value with error bars denoting standard error. (**b**,**c**) Results from each individual animal from each of the eight cage units. (**e**) Cumulative clinical score from all signs recorded throughout the study with each animal represented as a single point. *, *p* < 0.05.

The clinical disease progression was assessed in recipient animals dosed with Neumifil or vehicle and then housed adjacently to intranasally challenged donor animals. Donor animals were removed from the system 6 days post-challenge, as we have previously shown that no live virus was detected from respiratory samples at this timepoint [6], similar to early work establishing the hamster model of SARS-CoV-2 infection [7]. The weight loss in directly challenged animals was more rapid and sustained, whereas in both the vehicle and Neumifil groups there was a stabilisation and slight decline before animals returned to putting on weight from day 9, with no statistically significant difference between the vehicle and Neumifil groups (*p* > 0.05) (Figure 2a). The clinical disease was more severe in animals artificially inoculated through the intranasal route as compared to those acquiring infection through natural routes (Figure 2c). When the recipient groups were compared, those that received Neumifil had consistently lower clinical scores than those receiving the vehicle (Figure 2c), reaching significance 7.4 days post-challenge (*p* = 0.0117) and close to significance at 9 days post-challenge (*p* = 0.0587). When the cumulative clinical scores across the groups were compared (Figure 2e), there was a significant difference between the scores in the donor groups and the Neumifil and vehicle groups (*p* = 0.0009 and *p* = 0.0019, respectively). The difference between the recipient groups was also significant (*p* = 0.0046), demonstrating that the animals receiving Neumifil had lower cumulative clinical scores compared to those receiving vehicle. Results from the individual cage units showed variations in the weight and clinical score kinetics (Figure 2b,d, respectively) across the animals housed in the eight units.

**Figure 3:**
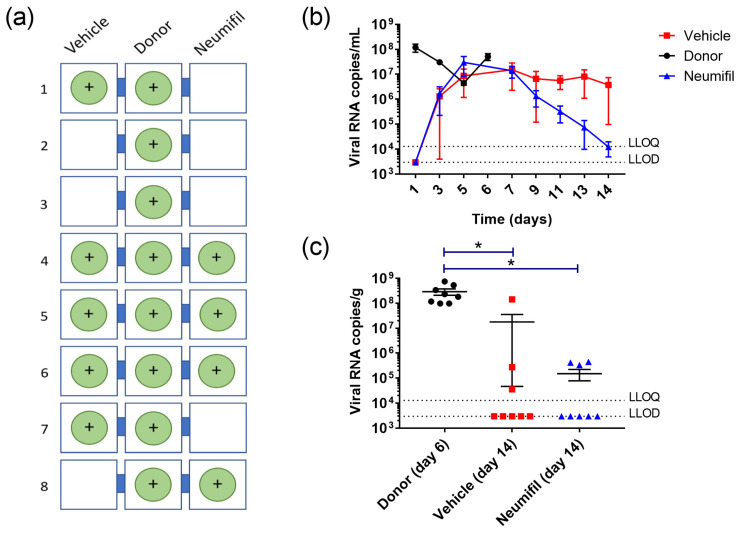
Viral RNA transmission and kinetics in donor animals and recipient animals receiving either Neumifil or vehicle. (**a**) Diagrammatic view of cages identifying the location of animals with PCR positive throat swab samples. (**b**) Levels of viral RNA detected in throat swabs. Symbols represent mean value, with error bars denoting standard error. No statistical significance was observed. (**c**) Levels of viral RNA in lung samples. Each animal represented is as a single point. *, *p* < 0.05.

Throughout the duration of the study, throat swabs were collected to assess viral RNA levels. Whilst all donor animals showed a positive PCR result from at least one throat swab, not all recipient animals did, with five out of eight (62.5%) recipient animals in the group receiving vehicle alone showing PCR evidence of infection (Figure 3a). Data from the throat swabs showed viral RNA levels in the recipient animals from day 3 onwards (Figure 3b). Levels in the recipient animals receiving Neumifil or vehicle remained similar until day 7, but thereafter appeared to reduce much more rapidly in the Neumifil group, although results did not reach statistical significance (*p* > 0.05). Within the lungs collected at necropsy (Figure 3c), there were significantly lower RNA levels in the recipient groups compared to the donor group (donor vs. vehicle, *p* = 0.008; donor vs. Neumifil, *p* = 0.005), although with different challenge kinetics and sample collection times the donor and recipient groups were not methodologically aligned, affecting direct comparison. There were no differences in lung RNA levels between the recipient groups (*p* = 0.9164) (Figure 3c).

**Figure 4:**
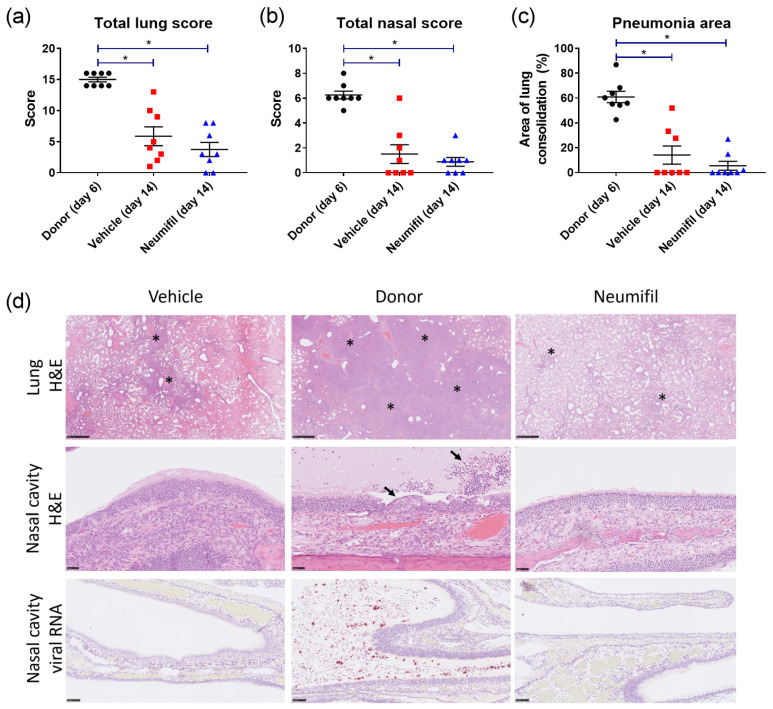
Histological readouts from donor or recipient animals treated with either Neumifil or vehicle after challenge with SARS-CoV-2. Total histopathology scores for (**a**) lung and (**b**) nasal cavity. (**c**) Percentage of area with consolidation (pneumonia) in the lung as determined by image analysis. (**a**–**c**) Individual animals are shown as a symbol, with line and whisker plots indicating the mean and standard error. *, *p* < 0.05. (**d**) Representative microscopic images of lung consolidation (top row, indicated by asterisks), changes in nasal cavity mucosa (middle row, indicated by arrows) and standard for SARS-CoV-2 RNA in the nasal cavity (lower row). Scale bars represent 1 mm (top row), 50 µm (middle row) and 100 µm (lower row).

Prominent lesions associated with SARS-CoV-2 infection were observed in the lung and nasal cavity of all animals in the donor group; in addition, viral RNA was detected in the nasal cavity (Figure 4d). By contrast, histological changes were less frequent and severe in the recipient groups, with viral RNA detected rarely in the nasal cavity. The findings suggest that there may be a slight decrease in the severity of pathological changes in the lung and nasal cavities of the recipient groups receiving a combination of prophylactic and therapeutic Neumifil treatment as compared to the vehicle recipient group. Histological lesions were scored to give a quantitative readout and the area of pneumonia was quantified, with results demonstrating increased severity in the donor animals compared to the recipient groups. This was statistically significant for lung score (Figure 4a; donor vs. vehicle, *p* = 0.0009; donor vs. Neumifil, *p* = 0.0009), nasal score (Figure 4b; donor vs. vehicle, *p* = 0.0033; donor vs. Neumifil, *p* = 0.0009) and the area of pneumonia (Figure 4c; donor vs. vehicle, *p* = 0.0014; donor vs. Neumifil, *p* = 0.0009). There were no statistically significant differences observed between the two recipient groups (*p* > 0.05).

## 4. Discussion

The clinical disease was more severe in animals artificially inoculated through the intranasal route as compared to those acquiring infection through natural routes (Figure 2c), with the differences likely attributable to different challenge doses which exert an effect upon disease progression [6,17].

The finding that only five out of eight (62.5%) recipient animals in the group receiving vehicle alone showed PCR evidence of infection (Figure 3a) was lower than expected. In work-up studies, we demonstrated 87.5% of recipient animals in this system having PCR positive results [6]. The group size chosen for this study was based on this level of infection, with the expectation that at least six recipient animals in the vehicle group would become infected, as this is the smallest number required for observing a significant 1-log reduction in viral load (based on a balanced one-way analysis of variance power calculation with a significance level of 0.05 and power of 0.8). Based on an infection rate of 62.5%, the future group size would need to be at least ten animals. In other studies looking at effects of SARS-CoV-2 natural transmission, group sizes of eight have also been applied, but with a difference of having animals pair-housed [8]. Due to previous observations of the transmission of SARS-CoV-2 between hamsters within the same cage [7], in our studies we have mitigated for this by singly housing for the duration. It has been shown that in the laboratory setting Syrian hamsters tolerate both social isolation and social housing conditions [18], and environmental enrichment was provided to ensure the welfare of the animals.

Comparing the clinical data (Figure 2d) and viral RNA detection (Figure 3a), some discrepancies in individual cages were detected. Whilst clinical signs were present in animals that tested positive for viral RNA, they were also reported in the remaining three animals in the vehicle group and an additional animal in the recipient group. However, the severity of clinical signs varied, with some animals just demonstrating ruffled fur whereas others displayed abnormal breathing. This could be because the viral RNA samples were based on throat swabs, rather than further down the respiratory tract where the virus might be more prevalent. Alternatively, for animals where only minor signs were recorded, the virus load might have been lower and not have breached the lower limit of detection of the RT-PCR assay.

In summary, our data demonstrate that Neumifil results in a significant reduction in the clinical disease severity of hamsters in a natural transmission setting. Results indicate a more rapid reduction of viral levels in throat swabs, indicative of clearance in the upper respiratory tract, but due to fewer animals becoming infected than predicted from work-up studies, statistical significance could not be achieved. This difference in infectivity levels is likely due to the outbred nature of the hamsters involved. Whilst transmissibility may also be affected by external factors, including temperature and humidity, these are controlled and regulated in the animal facility and it has been reported by others that environmental conditions do not overly affect the transmissibility of SARS-CoV-2; instead, it is extrinsically associated with the infectivity of the donor hamsters [19]. The use of different SARS-CoV-2 strains will also likely lead to variation in transmission efficiency, as reported for different variants in the hamster model [9,20]. Despite the limitations in the number of recipient animals becoming infected being lower than expected, the data presented within demonstrated that Neumifil exerts a significant reduction in clinical progression in a natural disease model, providing additional evidence to previous work which showed a positive effect after direct intranasal challenge [14]. Whilst this study was designed to test Neumifil administered as a combination of prophylactic and therapeutic uses, further experiments are warranted to ascertain timings and relevance for antiviral treatment initiation. Given the mechanism of action with targeting cellular receptors, and thus the applicability for a breadth of respiratory viruses, these types of compounds coming through development stages into clinical testing will extend the toolkit of interventions against current and future public health threats. In conclusion, we recognise that refinements of the natural transmission model applied in this study are required in order to ensure adequate transmission of the virus and allow for a thorough assessment of various candidate antiviral solutions, but also that the system has the potential to refine the preclinical testing of interventions using a challenge route which closely mirrors natural infection.

## Data Availability

The data presented in this article are available on request from the corresponding author.
